# Quantifying computational mechanisms in psychotherapy

**DOI:** 10.1192/j.eurpsy.2023.183

**Published:** 2023-07-19

**Authors:** Q. Huys

**Affiliations:** University College London, London, United Kingdom

## Abstract

**Abstract:**

Despite extensive research, the cognitive processes mediating the impact of psychotherapeutic interventions remain poorly understood, and as a result difficult to quantify. Identifying such mechanisms is likely to be extremely helpful: it could help target interventions better, could support dosing therapy through monitoring, and could heighten the speed at which new interventions can be developed. Mechanisms research in psychotherapy has described a number of key difficulties to achieving this. In this and the next talk, we ask whether advances in cognitive computational neuroscience might provide some support. Specifically, the question is whether precise cognitive probes might identify specific mechanisms of interventions. In support of this, I will first describe a pilot study in participants undergoing an adapted behavioural activation therapy. I will then move to present results from two strands of experiments examining whether interventions derived from components of cognitive-behavioural therapy (CBT) are able to shift computationally-derived measures of their proposed psychological substrates. Findings from both strands will be discussed with respect to challenges in developing brief, reliable, engaging, and user-acceptable measures of cognition. Overall, this outlines some early new results in using computational methods to understand therapeutic processes in the psychotherapy for depression.

**Disclosure of Interest:**

Q. Huys Grant / Research support from: Koa Health, Consultant of: Aya Technologies and Alto Neuroscience

